# SATB2 promotes radiation resistance of esophageal squamous cell carcinoma by regulating epithelial-to-mesenchymal transition via the Wnt/β-catenin pathway

**DOI:** 10.3389/fonc.2025.1543426

**Published:** 2025-02-26

**Authors:** Chen Lin, Youyi Wu, Yuchen Qian, Jiayi Li, Youdi He, Huang Yu, Congying Xie, Huafang Su

**Affiliations:** ^1^ Department of Radiation Oncology, The First Affiliated Hospital of Wenzhou Medical University, Wenzhou, China; ^2^ Zhejiang Key Laboratory of Intelligent Cancer Biomarker Discovery and Translation, First Affiliated Hospital of Wenzhou Medical University, Wenzhou, China; ^3^ Department Oncology Radiotherapy, The Third Affiliated Hospital of Wenzhou Medical University, Rui’an People Hospital, Ruian, Zhejiang, China

**Keywords:** SATB2, ESCC, radioresistance, Wnt/β-catenin signaling pathway, EMT

## Abstract

**Purpose:**

Radioresistance remains a predominant factor contributing to local recurrence in esophageal squamous cell carcinoma (ESCC). SATB2, as a transcriptional co-gene, may affect the radioresistance of cancer cells. Consequently, this study aims to elucidate the mechanism by which SATB2 modulates radiotherapy resistance in esophageal cancer.

**Methods:**

We identified highly expressed genes associated with radioresistance in ESCC using the MSigDB database and conducted survival correlation analysis. A radioresistant esophageal squamous cell carcinoma cell line (KYSE150R) was established using the gradient dose method, and RT-qPCR was used to detect the expression of SATB2 in KYSE150 and KYSE150R cells. CCK-8, Transwell, colony formation assay, and cell scratching were performed to determine and evaluate cell proliferation, cell migration, and cell invasion. Furthermore, the expression levels of mRNA and protein were correlated using WB and RT-qPCR. Mitochondrial membrane potential and apoptosis detection kits were used to evaluate the level of apoptosis. Finally, a mouse subcutaneous xenograft tumor model was employed to elucidate the role of SATB2 on the radiotherapy resistance of ESCC *in vivo*.

**Results:**

Bioinformatics analysis indicated that SATB2 is linked to increased drug resistance in esophageal cancer. The results demonstrated that suppression of SATB2 decelerates cell proliferation and migration, accelerates apoptosis, inhibits the GSK-3β (Ser9) phosphorylation, and reduces β-catenin and target gene C-myc. The addition of the Wnt/β-catenin signaling pathway agonist (CHIR-99021) reversed these effects. Xenograft studies in mice revealed that knockdown of SATB2 reduced ESCC radioresistance.

**Conclusion:**

We concluded that SATB2 may dysregulate the Wnt/β-catenin pathway, thereby facilitating EMT progression and conferring radioresistance.

## 1 Introduction

Esophageal cancer (1, 2) is a predominant global malignancy. The development of radioresistance following high-dose radiation therapy is a significant contributing factor to the unfavorable prognosis in esophageal cancer treatment (3). Consequently, uncovering the molecular mechanism of radiation tolerance in esophageal cancer types may offer critical insights into overcoming radiation tolerance.

SATB2 (4), also known as AT-rich DNA-binding protein 2, encompasses 11 exons spanning a 191-kb region on chromosome 2. The SATB2 protein is a crucial mediator of high-order chromatin architecture and gene expression regulation (5). Epithelial–mesenchymal transition (EMT) (6) is a focal point in oncological investigation, with its role in tumor radioresistance increasingly recognized (7). The Wnt/β-catenin signaling pathway (8) governs cellular proliferation and survival pathways. Dysregulation, through mutation and abnormal expression, of pathway components of this pathway can result in developmental defects and cancer onset. Recently, several studies have shown a functional interaction between SATB2 and the Wnt/β-catenin pathway, as well as its association with EMT (9–12).

In conclusion, our study reveals that overexpression of SATB2 can aberrantly activate the Wnt/β-catenin pathway, thereby inducing radioresistance and promoting EMT in esophageal cancer cells.

## 2 Materials and methods

### 2.1 Bioinformatics analysis

Four pathways associated with radioresistance were identified in the MSigDB database. Subsequently, radioresistance scores for each sample were calculated by using the ssGSEA package in version R (4.0.2). Utilizing these scores, we clustered all samples and obtained a heatmap, which fell into two clusters (potential high radioresistance and low radioresistance). We proceeded to analyze the expression differences of genes associated with the Wnt/β-catenin pathway between the two clusters, with the data distribution visualized using a vioplot. A PCA plot was then generated, illustrating the best separation between groups. Additionally, a volcano plot was created to depict the expression changes of all the genes, with a focus on those with | logFC > 6 |. Following this, both univariate and multivariate Cox analyses were conducted to identify differential genes based on grouping: high and low. Using the expression data of these differential genes, we constructed a risk model to evaluate the correlation between target genes and poor prognosis.

### 2.2 Cell culture

The human esophageal squamous cell carcinoma cell line KYSE150 was provided by the Type Culture Library, Chinese Academy of Sciences (Shanghai, China). For cultivation, these cells were maintained in RPMI 1640 medium (Gibco, United States) supplemented with 10% fetal bovine serum (Gibco, United States). The cells were incubated in a controlled environment within a cell culture incubator set to 37°C and 5% CO_2_.

### 2.3 Construction of the radiation resistance cell line (KYSE150R)

To establish a radiotherapy-resistant cell line, KYSE150 cells were monitored for their growth status. Cells in the logarithmic growth phase were selected and irradiated with 100 cGy/min dose rate of X-rays for linear acceleration at 6 MV. Following irradiation, the cells were promptly returned to a 37°C, 5% CO_2_ incubator. The cell culture was continued until growth reached 90%, at which point the cells were treated with trypsin (Beyotime, China) for digestion and subculturing. When the cell proliferation reached the exponential growth phase, it was irradiated at the same dose rate again. The above steps (100 cGy three times, 200 cGy three times, 400 cGy three times) were repeated to gradually establish the esophageal cancer radiotherapy-resistant cell line KYSE150R. Concurrently, the parental KYSE150 cells were cultured under identical conditions throughout the induction process but were not exposed to X-ray irradiation. After reaching a cumulative dose of 2,100 cGy, the radiation-resistant cell line KYSE150R was screened, and aliquots were stored in a −80°C refrigerator.

### 2.4 Cell transfection

For transient transfection, the KYSE150R cell lines were transfected when they reached a cell density of 50%–70% using Lipofectamine 2000 (Invitrogen, USA). Si-SATB2 and its negative control were provided by Gene Pharmaceuticals (GenePharma, China), resulting in the creation of a SATB2 gene knockdown group and a no-load group. The cells were first starved for 30 min. Following the preparation of the transfection mixture, the mixed transfection solution was added to the starved cells according to different groups and gently placed in an incubator for culture. After an incubation period of 4–6 h, the transfection reagent containing the culture medium was discarded, and 2 mL of the complete culture medium supplemented with 10% serum was added to each well. The cells were then cultured under normal conditions at 37°C and 5% CO_2_ for 2 days. The specific siRNA sequences are detailed in **Table 1**. For stable transfection, Sh-SATB2 was designed based on the gene sequence of Si-SATB2, which had the highest transfection efficiency in transient transfection. The viral vector used in this study was named SATB2 shRNA in pLenti-U6-shRNA-CMV-GFP-2A-Puro (human); the Sh-SATB2 sequence was 5′-CCAGCGTCCAATGCATTTATTCAAGAGATAAATGCATTGGACGCTGG-3′, with a total length of 47 bp (RiboBio, China). To establish the optimal concentration of puromycin (RiboBio, China) for selection, the final concentration was determined as the lowest concentration at which complete cell death was observed, which served as the ideal concentration for generating puromycin-resistant cells. The final concentration of puromycin for KYSE150R cells was found to be 10 μg/mL. KYSE150R cells in good condition and in the logarithmic growth phase were then seeded into 96-well plates to determine the optimal infection multiplicity of infection (MOI) value. The determined optimal MOI value for this experiment was 20. Using the optimal MOI value from the preliminary experiment, a fresh complete medium was employed to dilute the virus to the required concentration, with polybrene (RiboBio, China) added at a final concentration of 5 μg/ml. The original culture medium of the cells was added and the diluted virus solution was also added based on the MOI value of the cells. Twenty-four hours post-infection, the culture medium containing the lentivirus was removed and replaced with a fresh culture medium.

**Table 1:** The Si-SATB2 sequence.

Si-SATB2	Sequences (5′–3′)	
SATB2-homo-2271	Sense	GGAUCCUCCAAAGCUUUAUTT
	Antisense	AUAAAGCUUUGGAGGAUCCTT
SATB2-homo-912	Sense	CAGUCCGCAAUGCCUUAAATT
	Antisense	UUUAAGGCAUUGCGGACUGTT
SATB2-homo-1138	Sense	CCAGCGUCCAAUGCAUUUATT
	Antisense	UAAAUGCAUUGGACGCUGGTT

### 2.5 RT-qPCR

A TRIzol reagent (Invitrogen, USA) was utilized to get the total RNA. The genomic DNA from the previously extracted total RNA was removed and the following mixture was prepared in an enzyme-free centrifuge tube: 4 μL of 4× gDNAwiperMix + 1 μg total RNA, followed by the addition of enzyme-free water to a final volume of 16 μL, and the mixture was gently mixed using a pipette. The mixture was then placed in a reverse transcription instrument and incubated at 42°C for 2 min. The reverse transcription reaction mixture was prepared in an RNase-free centrifuge tube (the mixture in the previous step + 4 μL 5×HiScript II qRT SuperMix II), and the above mixture was stirred evenly. A reverse transcription reaction was performed, the program was run at 50°C for 15 min and 85°C for 5 s, and cDNA was added. The SYBR qPCR Master Mix kit (Vazyme, China) was used to measure mRNA levels of the target gene. The calculation formulae were as follows: ΔCt = Ct target gene − Ct GAPDH; ΔΔCt = ΔCt experimental group − ΔCt control group; relative expression amount of the target gene = 2−ΔΔCt. The primer sequences are shown below (**Table 2**).

**Table 2:** Primer sequences.

Genes	Primer	Sequences (5′–3′)
GAPDH	Sense	GGTGGTCTCCTGTGACTTCAA
	Antisense	CCACCCTGTTGCTGTAGCC
SATB2	Sense	CCAAACACACCATCATCAAGTTCTTC
	Antisense	CCTCGCTGTCGTTCTCCTCTG
Bcl-2	Sense	TGTGGATGACTGAGTACCTGAACC
	Antisense	CAGCCAGGAGAAATCAAACAGAGG
Bax	Sense	CATGGGCTGGACATTGGACTTC
	Antisense	CACAAAGATGGTCACGGTCTGC
E-cadherin	Sense	ATTTTTCCCTCGACACCCGAT
	Antisense	TCCCAGGCGTAGACCAAGA
Slug	Sense	TCGGACCCACACATTACCTT
	Antisense	TGACCTGTCTGCAAATGCTC

### 2.6 Western blot analysis

The concentration of the SDS-PAGE gel was chosen based on the molecular weight of the protein, and total protein was then separated using SDS-PAGE and transferred to a PVDF membrane (Millipore, USA). Following 2 h of incubation for slow sealing on a shaker at room temperature, the diluted antibody was added and incubated overnight at 4°C. Subsequently, the membrane was removed and washed, and the secondary antibody (Biosharp, China) was added and incubated on a shaker at room temperature for 1 h. The protein band image was then acquired through film exposure (Fujifilm, Japan) using an ECL exposure solution (Thermo, United States). The sources of the antibodies are listed in **Table 3**.

**Table 3:** The antibodies’ sources.

Antibodies	Sources
SATB2	Abcam, USA
E-cadherin	Cell Signaling Technology, USA
Slug	Cell Signaling Technology, USA
β-Catenin	Cell Signaling Technology, USA
C-myc	Cell Signaling Technology, USA
GSK-3β	Cell Signaling Technology, USA
p-GSK-3β (Ser9)	Cell Signaling Technology, USA
Bcl-2	Proteintech, China
Bax	Proteintech, China
β-Actin	Proteintech, China
Histone H3	Proteintech, China

### 2.7 Scratch test

A black permanent marker was used to inscribe horizontal lines on the exterior bottom surface of a six-well plate, ensuring five parallel lines per well, primarily to facilitate accurate positioning during photography. The cells were digested in the logarithmic growth phase using trypsin, and the cell passage procedure was performed. The cells were seeded into a six-well plate that was marked with lines, and the plate was gently rocked using a cross-motion technique and incubated at 37°C with 5% CO_2_ for standard culture conditions. Once the cells reached 80% confluence, the culture medium was removed, and a 1,000-μL pipette tip was used, held perpendicular to the bottom of the six-well plate to draw lines perpendicular to the pre-existing black lines. An RPMI 1640 culture medium was added supplemented with 2% fetal bovine serum and incubated under standard culture conditions. Images were captured using an optical microscope (Olympus, Japan) at 0 h, 12 h, and 24 h post-lineation. The scratch area was quantified using ImageJ and analyzed using the Prime software.

### 2.8 Transwell invasion assay

Seven hundred microliters of culture medium was added to the lower chamber and placed within a small incubation chamber, and then 400 μL of the same medium was added to the upper chamber and incubated at 37°C with 5% CO_2_ for 15 min. The medium from both the upper and lower chambers was aspirated, 700 μL of complete culture medium supplemented with 10% fetal bovine serum was added to the lower chamber, and 300 μL of a single-cell suspension was introduced, prepared in a serum-free medium, into the upper chamber [precoated with Matrigel (BD, USA)], inoculating 50,000 cells per well. Subsequently, the setup was incubated at 37°C with 5% CO_2_ for 24 h. Paraformaldehyde (4%) was added to fix the cells. Following a 30-min fixation period, the formaldehyde was removed, and the cells were stained with 0.5% crystal violet solution for 10 min and subsequently discarded. Once the upper chamber had dried, cell invasion was visualized using an inverted optical microscope.

### 2.9 CCK-8 assay

Cells were irradiated at 0, 2, and 8 Gy, and the standard set by the machine was 200 cGy/min X-ray at a linear rate of 6 MV. Subsequently, cells were seeded into 96-well plates (1 × 10^3^ cells/well) and treated with the CCK-8 kit (MCE, USA) following cell attachment (0 h) and 24, 48, and 72 h post-culture. Using an enzyme-linked immunoassay (TECAN, Switzerland), absorbance at 450 nm was measured for each well, enabling the assessment of cell proliferation changes based on absorbance values. The calculation formula was as follows: X-hour cell viability = X-hour absorbance of the experimental group − X-hour absorbance of the blank group/0-hour absorbance of the experimental group − 0-hour absorbance of the blank group.

### 2.10 Clonogenic assay to determine cellular radiosensitivity

X-rays at doses of 0, 2, 4, 6, and 8 Gy were used to irradiate the cells, where the standard set by the machine was a linear acceleration of 6 MV (200 cGy/min). Subsequently, the cells were seeded into a six-well plate (100–500 cells/well). Following a 7–10-day culture period, the cells were fixed for 30 min in 4% polymethyl methacrylate (Beyotime, China) and then stained with 0.2% crystal violet solution (Beyotime, China) at 37°C for 10 min. After imaging the cell colonies using a mobile phone, the number of colonies in each group was quantified with ImageJ software, and the cell survival curve was constructed using a multitarget single-click model. Subsequently, the radiotherapy sensitivity of the cells following different treatments was determined.

### 2.11 Cell viability and apoptosis analysis

The cell culture dish was centrifuged for 5 min with a 1,000*g* centrifuge, suitable for a porous plate. The cell culture medium was aspirated, and the cells were washed once with PBS (Beyotime, China). The mitochondrial membrane potential kit (Beyotime, China) was utilized to assess cell viability and apoptosis. To each well, 188 µL of Annexin V-FITC binding solution and 5 µL of Annexin V-FITC were added, followed by gentle mixing. Subsequently, 2 μL of Mito-Tracker Red CMX Ros staining solution and 5 μL of Hoechst 33342 staining solution were added, and the mixture was gently agitated again. The samples were incubated at room temperature (20°C–25°C) in the dark for 20–30 min. Subsequently, fluorescence expression was observed using a fluorescence microscope (Nikon, Japan).

### 2.12 Animal model

Twenty-four male BALB/C-nu/nu nude mice were acquired and approved by the ethics committee (WYYY-IACUC-AEC-2024-012). The groups were as follows: KYSE150R+Sh-SATB2 group, KYSE150R+NC group, KYSE150R+Sh-SATB2+IR group, and KYSE150R+NC+IR group (*n* = 6). Each mouse was subcutaneously injected with 100 μL. Every 3 days, the size of the subcutaneous xenograft was recorded. Upon reaching a volume of 80 mm^3^, six were selected from each of the SATB2 knockdown group and the negative control group for radiotherapy (total dose 8 Gy in two separate exposures). The nude mice were killed by a high concentration of CO_2_ and the xenografts were harvested after 32 days. Following the removal of the xenografts, the tumors were photographed and weighed, and their dimensions were calculated.

### 2.13 TUNEL assay

The tissue was embedded in paraffin, sectioned, and dehydrated. The slices were dried at 67°C for 30 min. The paraffin sections were dewaxed, and proteinase K (Beyotime, China) working solution was added to cover the tissue and incubated at room temperature for 20 min at 37°C. After the sections had partially dried, the membrane permeabilization working solution was applied to cover the tissue and incubated at room temperature for 10 min. The appropriate volume of TdT and Lable Solution (Beyotime, China) was taken based on the number of sections and tissue size and mixed in a 1:9 ratio. The tissue was covered with the mixture. The sections were placed flat in a humidity chamber and incubated in a water pot at 37°C for 60 min, and periodically, a small amount of water was added to the chamber to maintain humidity. Once the sections had partially dried, 50 μL of DAPI stain solution (Beyotime, China) was added to the area of interest and incubated at room temperature in the dark for 5 min. Following partial drying of the sections, they were sealed with an antifluorescence quenching sealing agent, and three fluorescence images of each sample were taken to calculate the cell apoptosis rate.

### 2.14 Immunohistochemical and immunofluorescent staining

The paraffin-embedded sections obtained *in vivo* were dewaxed and incubated in citric acid buffer (Beyotime, China) at boiling temperature for 10 min for antigen recovery. Endogenous peroxidase activity was quenched, and the sections were incubated with the primary Ki-67 antibody (1:100, Roche, Basel, Switzerland) overnight. Secondary antibodies were added to the tissue surface (Santa Cruz, USA). Diaminobenzidine (DAB, Meryer, China) and hematoxylin staining was conducted for 3 min. Following dehydration through a gradient of ethanol, the sections were sealed with neutral resin (Beyotime, China). Subsequently, the image of the stained section was obtained using a light microscope.

### 2.15 Statistical analysis

GraphPad Prism6 was selected to analyze the data obtained from the experiment, where the mean ± standard deviation (SD) was used to represent the analyzed data. The two-tailed unpaired *t*-test was used between the two groups, and one-way ANOVA was used in more than three groups. Statistical analysis showed that there was statistical significance (*p* < 0.05).

## 3 Results

### 3.1 SATB2 is a highly expressed gene related to radioresistance in esophageal cancer

**Figure 1:**
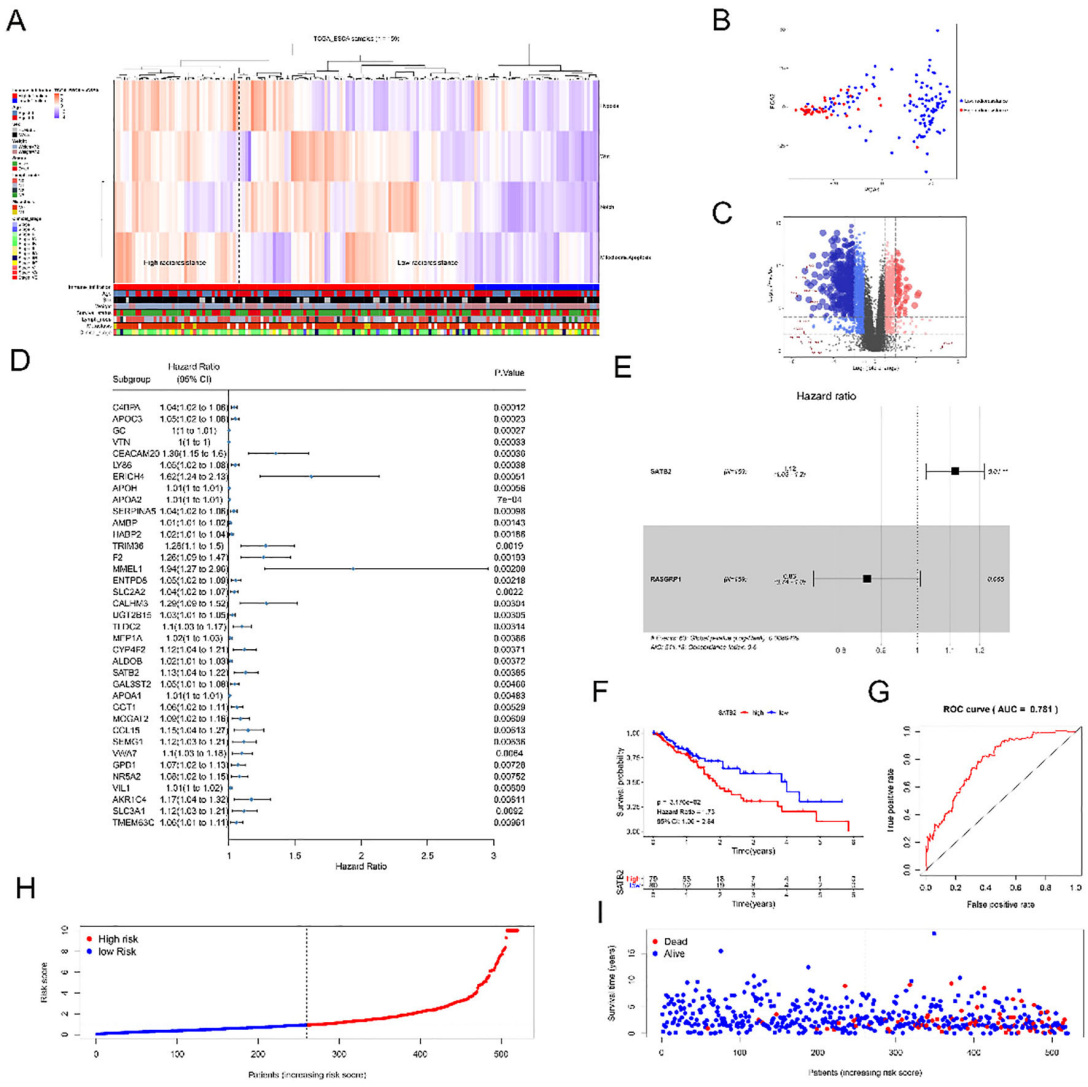
SATB2 affects radioresistance of ESCC cells. **(A)** Clustering result map of gene set of signal Pathway in esophageal Cancer samples in TCGA Database. **(B)** Principal component analysis of genes of high radioresistance and low radioresistance. **(C)** Volcano diagrams of all sample genes andlog2FC> 6 genes. **(D)** Univariate Cox analysis of 37 genes. **(E)** Multivariate Cox analysis of SATB2 and RASGRP1. **(F)** Risk model of SATB2 with different levels of expression. **(G)** ROC curve analysis of the value of SATB2 in evaluating survival and prognosis. **(H)** The distribution of risk score in patients. **(I)** The distribution of survival time in patients.

To identify differentially expressed genes between the high-radioresistance and low-radioresistance groups, we identified four radioresistance-related signal pathways in the MSigDB database: 1) hypoxia-inducible factor 1 alpha signal pathway, 2) positive regulation of the canonical Wnt signal pathway, 3) positive regulation of the Notch signal pathway, and 4) apoptotic mitochondrial changes. The gene set enrichment scores for the four signaling pathways in each sample were determined using ssGSEA analysis. Based on the signaling pathway gene set, the samples were classified into potential high- and low-radioresistance groups (**Figure 1A**). Subsequently, we conducted principal component analysis on all genes within the two datasets to identify differentially expressed genes between the high- and low-radioresistance groups (**Figure 1B**). Additionally, we visualized all genes using a volcano plot (**Figure 1C**). There were 2,021 screened differentially expressed genes obtained (the genes listed were | log2FC | > 6). Subsequently, we subjected the 2,021 screened differentially expressed genes (DEGs) to univariate Cox analysis and identified 37 genes that were significantly associated with survival (*p* < 0.0l) (**Figure 1D**). Only two genes, SATB2 and RASGRP1, were found to correlate with survival in the Kaplan–Meier test. Consequently, we selected SATB2 and RASGRP1 for multivariate Cox analysis (**Figure 1E**), which revealed that only SATB2 was statistically significant. Additionally, we developed risk models based on high and low SATB2 expression (**Figure 1F**). Compared with the high-risk group, the viability of the low-risk group was significantly higher (*p* < 0.01). The accuracy of this survival model was assessed using ROC curves (**Figure 1G**), with results indicating statistical accuracy and reliability (AUC = 0.781). Utilizing the constructed model, we employed survStat to describe the distribution of risk scores. The higher the risk score of the patients, the higher the risk score of the corresponding model (**Figure 1H**) and the higher the mortality rate (**Figure 1I**). In conclusion, SATB2 is markedly elevated in the high-risk groups. Consequently, SATB2 was identified as the biomarker influencing the radiosensitivity of ESCC for subsequent experimental investigations.

### 3.2 SATB2 exists in KYSE150R cells with higher cell viability and epithelial–mesenchymal transition ability

**Figure 2:**
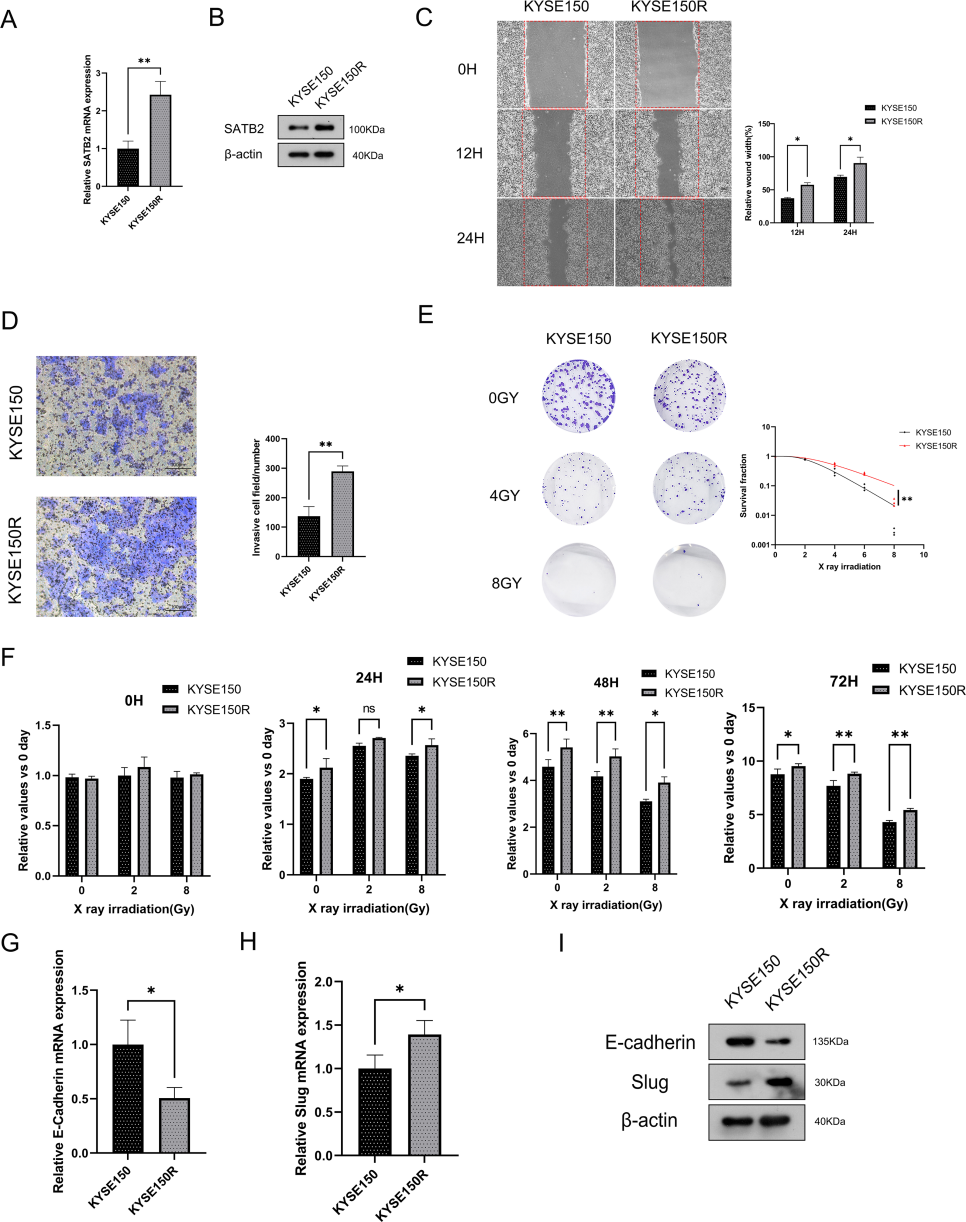
SATB2 exists in KYSE150R cell with higher cell viability and EMT ability. **(A, B)** SATB2 expression levels were measured at the mRNA and protein levels. **(C, D)** Assessment of cell migration rate and invasion ability. **(E)** The survival of these two kinds of cells after X-ray irradiation was analyzed by using a multitarget single-shot model. **(F)** The two kinds of cells were irradiated by X-ray and the activity of the two kinds of cells was determined by CCK-8. **(G–I)** RT-qPCR to evaluate gene expression in cells, and then Western blotting was used to further verify these proteins’ expression. *p < 0.05, **p < 0.01 vs. KYSE150.

To investigate the impact of SATB2 on radioresistance in esophageal cancer cells, we developed radioresistant esophageal cancer cells (KYSE150R cells). This cell line exhibited radioresistance, resulting in a limited response to radiotherapy. We confirmed that SATB2 is indeed overexpressed in KYSE150R cells (**Figures 2A, B**). Additionally, through cell scratching and Transwell assays, we observed that KYSE150R cells significantly outperformed the KYSE150 (human esophageal cancer epithelial cell line) in terms of cell migration (**Figure 2C**) and invasion (**Figure 2D**). Moreover, we demonstrated that the survival rate of KYSE150R cells was significantly higher than that of the parent cell lines across various radiation doses (**Figure 2E**). Subsequently, CCK-8 assays revealed that KYSE150R cells exhibited enhanced proliferation capabilities, irrespective of the irradiation dose (**Figure 2F**). In the final set of experiments, we performed RT-qPCR and WB on the KYSE150 and KYSE150R cells. Our results found that, in KYSE150R cells, E-cadherin (EMT-related molecule) was decreased, while the expression of slug in KYSE150R cells was increased (**Figures 2G–I**). These results suggest that KYSE150R cells may exhibit enhanced radioresistance through the promotion of EMT, and SATB2 is likely involved in the regulation of these processes.

### 3.3 Silencing SATB2 can reduce radioresistance and EMT ability of KYSE150R cells and promote apoptosis

**Figure 3:**
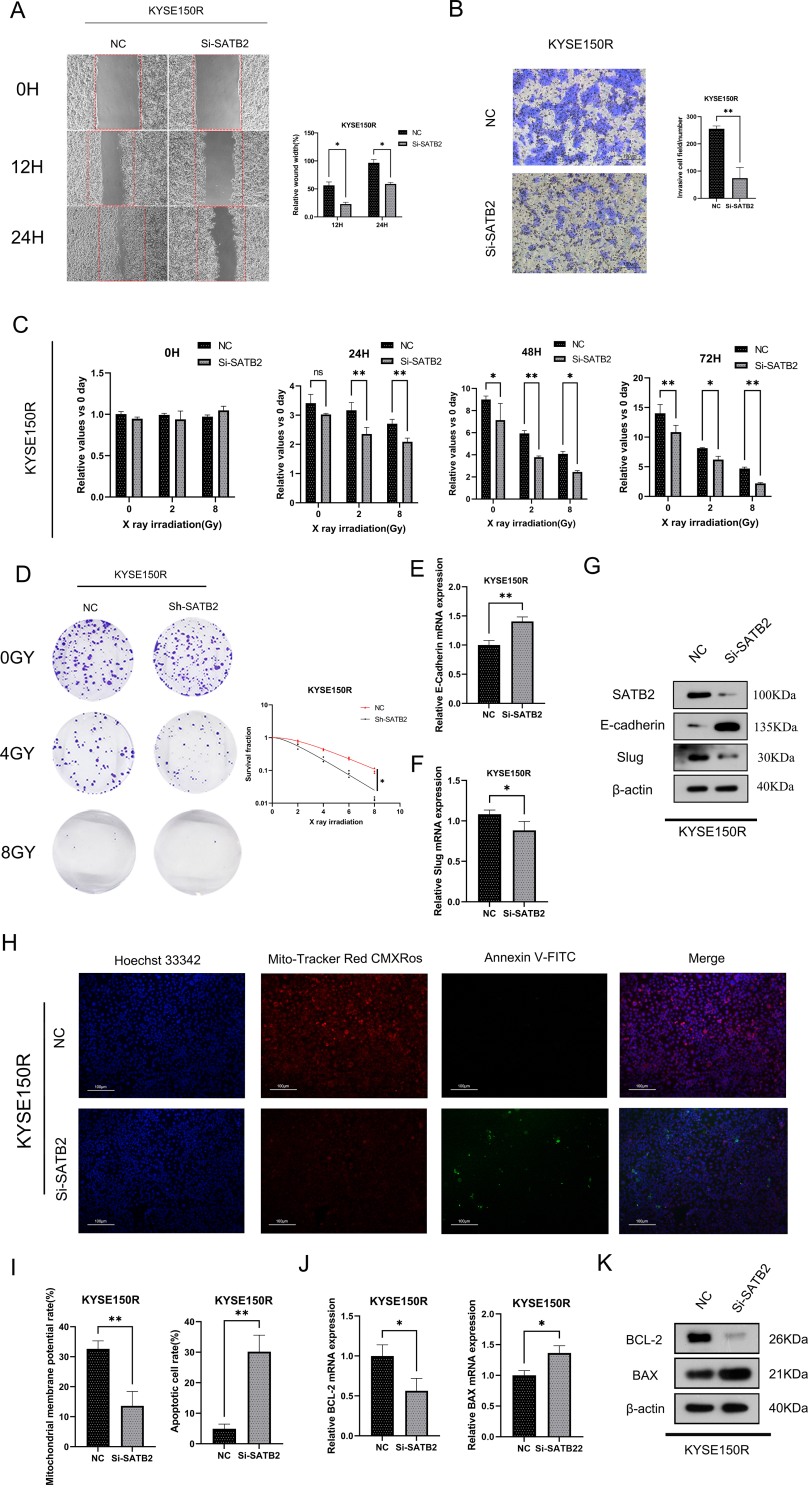
Knockdown of SATB2 can impair the radioresistance and EMT capacity of esophageal cancer cells. **(A–C)** Changes obtained in KYSE150R cell migration, invasion ability, and cell viability after silencing SATB2. **(D)** KYSE150R cells were stably silenced by different doses of X-ray irradiation after SATB2. Using the colony formation test to analyze cell viability and detect the changes in radiation resistance of cells after different treatments. **(E, F)** After silencing SATB2, changes in E-cadherin and slug were detected in cells. **(H, I)** After silencing SATB2. Detection of cell viability and stability of cell membrane potential by fluorescence probe (×5). **(J, K)** After silencing SATB2. Using RT-qPCR and Western blotting to assess the expression of apoptotic protein. *p < 0.05, **p < 0.01 vs. NC.

After transfecting three distinct Si-SATB2 sequences into KYSE150R cells, we detected by WB that the sequence with the highest protein knocking efficiency was SATB2-homo-1138 (**Supplementary Figure S1**). Hence, this sequence was selected for the subsequent experiments. The transfection of Si-SATB2 or Sh-SATB2 (lentiviral vector) to inhibit SATB2 expression in KYSE150R cells resulted in significantly impaired invasion and migration of the cells following Si-SATB2/Sh-SATB2 treatment (**Figures 3A, B**). Additionally, the most significant reduction in the viability of KYSE150R cells after SATB2 silencing was most pronounced under X-ray irradiation at a dose of 8 Gy (**Figure 3C**). Moreover, the silencing of SATB2 significantly decreased the survival fraction of KYSE150R cells across various irradiation doses (**Figure 3D**). Studies (14, 15) indicated that EMT served as a marker of tumor metastasis and was implicated in the processes associated with radioresistance in cancer. Our findings revealed that inhibiting SATB2 expression decelerated the EMT process in the cells (**Figures 3E-G**). The study concluded that the expression of red fluorescence in KYSE150R cells diminished following SATB2 silencing, suggesting that silencing of SATB2 attenuated cell viability. A higher number of green fluorescence cells compared to the negative control group indicated that silencing of SATB2 promoted apoptosis (**Figures 3H, I**). Furthermore, the silencing of SATB2 inhibited BCL-2 expression and enhanced BAX expression (**Figures 3J, K**). The aforementioned experimental findings collectively indicated that SATB2 facilitated the progression of EMT, thereby enhancing radioresistance in esophageal cancer cells.

### 3.4 SATB2 promotes radioresistance of KYSE150R cells by means of the Wnt/β-catenin pathway

**Figure 4:**
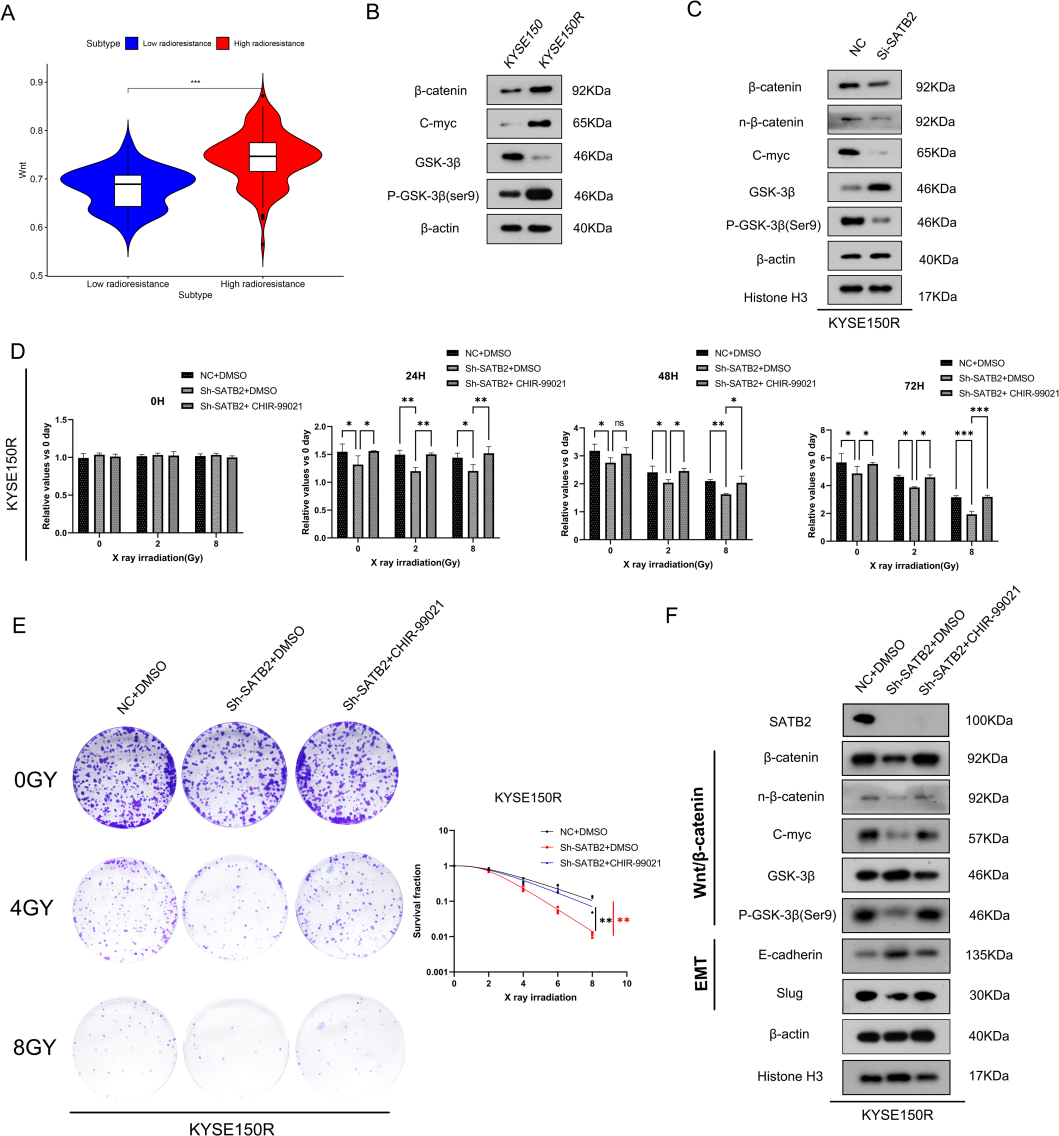
Silencing SATB2 reduces the radioresistance and EMT in KYSE150R cells via the Wnt/β-catenin pathway. **(A)** Vioplot of clustering results in the Wnt/β-catenin signaling pathway. **(B)** The vital molecules of the Wnt/β-catenin signaling pathway in cells were observed. **(C)** Using Western blot to detect the activation of Wnt/β-catenin in KYSE150R cells after silencing SATB2. **(D–F)** KYSE150R cells after silencing SATB2 were treated with CHIR-99021 for 24 **(h)** After receiving different doses of X-rays, the CCK-8 test showed the ability of cell proliferation, the plate cloning test detected the survival fraction in each group, and Western blot determined the changes in the Wnt/β-catenin signaling pathway and EMT progression. *p < 0.05, **p < 0.01, ***p < 0.001; ns, no significance.

After obtaining the aforementioned results, we aimed to elucidate the mechanism by which SATB2 regulates radioresistance and EMT in ESCC cells. Certain studies (13, 16, 17) have demonstrated that overexpression of SATB2 leads to dysregulation of the Wnt/β-catenin signaling pathway. The data distribution in the vioplot indicated significant differences in the Wnt/β-catenin pathway between the high and low radiation resistance groups (**Figure 4A**). As depicted in **Figure 4B**, an abnormal increase in the activity of the Wnt/β-catenin signaling pathway was observed in KYSE150R cells compared to KYSE150 cells. Additionally, downregulation of SATB2 expression inhibited GSK-3β phosphorylation, which consequently reduced β-catenin production. Moreover, silencing SATB2 decreased nucleus β-catenin expression and the expression of C-myc (β-catenin-targeted gene) in KYSE150R cells (**Figure 4C**). CHIR-99021, an aminopyrimidine derivative, functions by inhibiting GSK-3β activity. It is also regarded as an effective activator of the Wnt/β-catenin signaling pathway. Therefore, we employed CHIR-99021 for rescue experiments to further substantiate that SATB2 influences the radioresistance of KYSE150R cells via the Wnt/β-catenin signaling pathway. After stably silencing SATB2, we added CHIR-99021 for 24 h (5 μM) to conduct the subsequent experiment. CCK-8 and plate cloning assays indicated that CHIR-99021 could rescue the inhibition of cell viability and radiation resistance in KYSE150R cells induced by silencing SATB2 (**Figures 4D, E**). Moreover, in comparison to the sh-SATB2 group, the expression of GSK-3β was reduced in the Sh-SATB2+CHIR-99021 group, whereas the levels of C-myc, β-catenin, and nuclear-β-catenin were elevated. Concurrently, it was also found that the EMT process was enhanced, and SATB2 may serve as a target site that can activate the Wnt/β-catenin signaling pathway to control the cell EMT process (**Figure 4F**). Based on the aforementioned experiments, we deduced that silencing of SATB2 mitigated the EMT and radioresistance in KYSE150R cells, attributable to the suppression of the overactive Wnt/β-catenin signaling pathway.

### 3.5 Downregulation of SATB2 can affect the growth and radioresistance of ESCC cell xenografts *in vivo*

**Figure 5:**
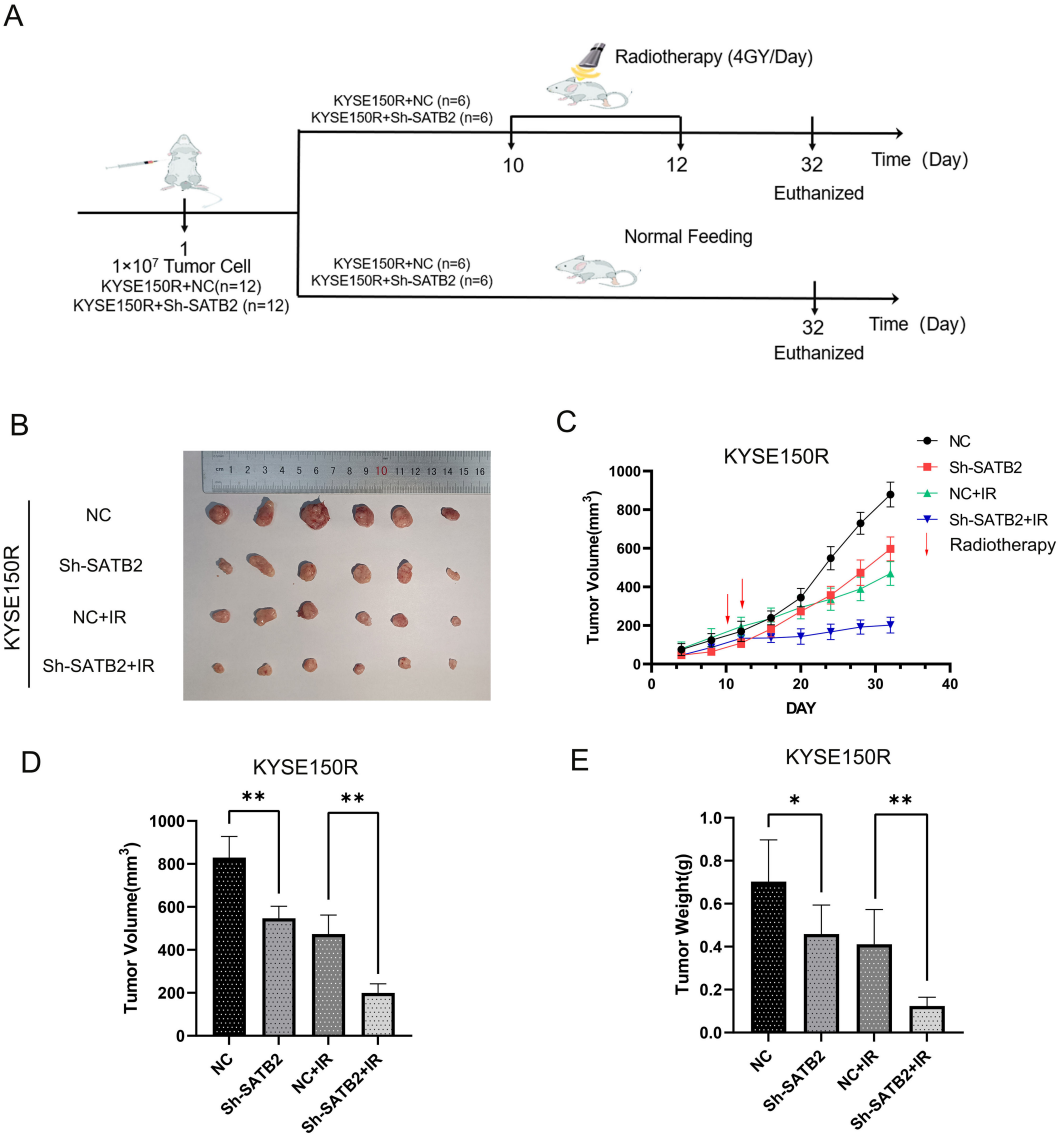
Effect of stably expressed low SATB2 on xenografts *in vivo*. **(A)** Chart of the mouse modeling process. **(B)** Macroscopic view of tumor tissue in experiments *in vivo*. **(C)** The growth curve of tumor tissue after treatments. **(D, E)** Analysis of tumor tissue after dissection. *p < 0.05, **p < 0.01 vs. NC/NC+IR. *p < 0.05 vs. NC, ** *p* < 0.01 vs. NC/NC+IR.

To assess radioresistance in esophageal cancer following the stable downregulation of SATB2 *in vivo*, we developed a BALB/C-nu/nu nude mouse ESCC xenograft model. The xenografts of nude mice were irradiated with X-rays (single dose 4 Gy) on the 10th and 12th day after injection of tumor cells, respectively (**Figure 5A**). We observed the most significant increase in tumor volume in the NC group, accompanied by a marked reduction in mouse activity. Tumors with stably reduced SATB2 expression (Sh-SATB2+IR) that received subsequent radiotherapy exhibited the slowest growth rate. After the treatment, the tumor tissues in each group were excised. The tumor tissues were solid and firm in texture. Notably, compared to the NC group, the tumors with stably reduced SATB2 expression (Sh-SATB2 group) were significantly smaller. More importantly, following radiotherapy, the Sh-SATB2+IR group demonstrated a more pronounced reduction in size compared to the NC+IR group (**Figures 5B, C**). Subsequent measurement of the weight and volume of tumor tissue confirmed that radiotherapy after SATB2 silencing significantly inhibited tumor growth (**Figures 5D, E**). The tumor inhibition rate was calculated (**Table 4**). The results indicated that the tumor inhibition rate (both volume and weight) of the Sh-SATB2+IR group (vs. the Sh-SATB2 group) was more obvious than that of the NC+IR group (vs. the NC group), with a volume inhibition rate of 66.88% and a mass inhibition rate of 73.03%. These findings suggested that the radioresistance of tumor tissue is markedly diminished after SATB2 knockdown, confirming that SATB2 can indeed influence the radioresistance of ESCC tissue *in vivo*.

**Table 4:** Mass tumor inhibition rate.

Group	Tumor volume (mm^3^)	Tumor weight (g)	Volume inhibition (%)	Weight inhibition (%)
NC	730.14 ± 225.83	0.70 ± 0.18	0	0
Sh-SATB2	581.98 ± 103.58	0.46 ± 0.12	0	0
NC-IR **(vs. NC)**	481.87 ± 203.45	0.41 ± 0.145	34%	41.56%*
Sh-SATB2+IR **(vs. Sh-SATB2)**	192.74 ± 60.59	0.12 ± 0.04	66.88%^#^	73.03%^##^

*p < 0.05 vs. NC, ^#^ *p* < 0.05, ^##^ *p* < 0.01 vs. NC+IR.

### 3.6 Stably low expressed SATB2 attenuates xenograft radioresistance by inhibiting the Wnt/β-catenin signaling pathway

**Figure 6:**
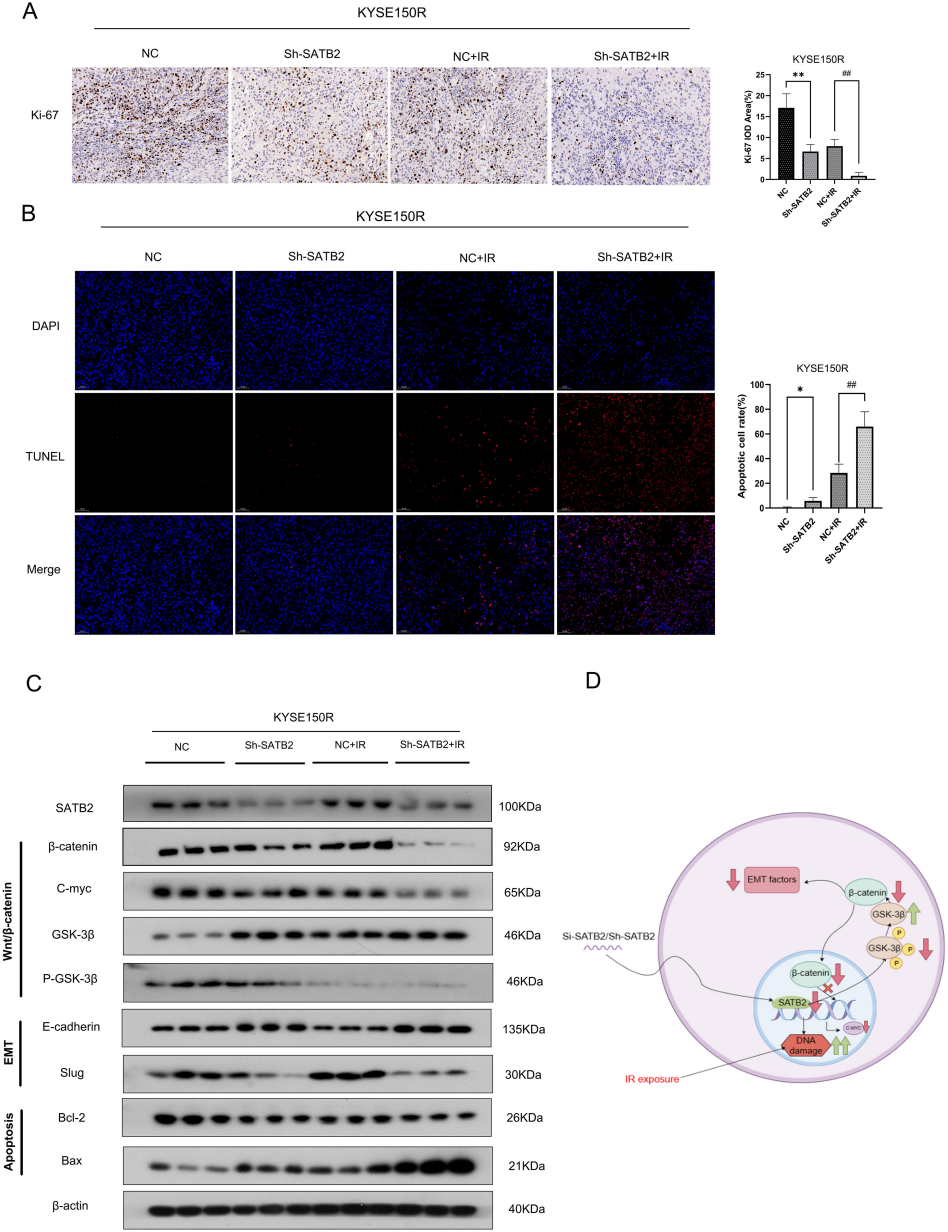
Mechanism of silencing SATB2 in weakening radioresistance of xenografts *in vivo.* **(A)** Detecting the level of Ki-67 to evaluate the proliferative activity of tumor cells (×20). **(B)** TUNEL assay to detect and analyze apoptosis in tumor tissues (×20). **(C)** Differences in the expression of the EMT phenotype and apoptosis in tumor tissues, as well as activation of the Wnt/β-catenin pathway. **(D)** Carding diagram of all the mechanisms in this study. IR, infrared radiation. *p < 0.05, **p < 0.01 vs. NC, ^##^ *p* < 0.01 vs. NC+IR.

The mechanism by which stably lowly expressed SATB2 regulates radioresistance in esophageal cancer tissues requires further investigation, and we found that among all treatments, tumor tissues in which SATB2 was silenced exhibited the lowest Ki-67 expression following radiotherapy (the Sh-SATB2+IR group) (**Figure 6A**). Concurrently, the TUNEL assay revealed a significant increase in the apoptosis rate within tumor tissues following SATB2 silencing and radiotherapy (the Sh-SATB2+IR group) (**Figure 6B**). Furthermore, we confirmed that EMT was inhibited in the group with stably low SATB2 expression, with the inhibition being more pronounced in the Sh-SATB2+IR group, which underwent both SATB2 silencing and radiotherapy. More importantly, we found that in the tumor tissues, the inhibition of the Wnt/β-catenin signaling pathway was the most obvious following SATB2 silencing and radiotherapy (the Sh-SATB2+IR group). Moreover, we also found that apoptosis was significant in tumor tissues in the Sh-SATB2+IR group following SATB2 silencing and radiotherapy (**Figures 6C, D**). These findings suggested that stable low expressed SATB2 inhibits EMT and weakens the radioresistance of xenografts *in vivo* and enhances apoptosis by suppressing the overactivation of the Wnt/β-catenin signaling pathway.

## 4 Discussion

Previous studies (5, 13, 14) have demonstrated that uncontrolled SATB2 expression can induce a malignant phenotype in cells, rendering it a potential marker of metastasis. Nevertheless, the impact of SATB2 on radioresistance has yet to be thoroughly investigated in ESCC cells. Hence, this research was conducted mainly with a view to delineate the role of SATB2 in the regulation of ESCC radioresistance and its molecular mechanism.

In our research, we successfully established a radioresistant cell line (KYSE150R) and found that the invasion, migration, cell viability, and radioresistance of the KYSE150R cells were much better than those of the parent cell line KYSE150 cells. Furthermore, numerous studies have indicated that the dysregulation of SATB2 is in connection with the malignant progression of cancer cells. Unsurprisingly, compared with KYSE150 cells, we observed that SATB2 was overexpressed in KYSE150R cells. Therefore, we silenced the expression of SATB2 in KYSE150R cells to investigate whether this knockdown altered the phenotypic characteristics of KYSE150R cells. Finally, our findings indicated that silencing of SATB2 not only weakened the migration, invasion, and viability of KYSE150R cells but also diminished their survival rate in response to radiation. The results implied that silencing of SATB2 concurrently suppresses the phenotype and radioresistance of KYSE150R cells.

EMT (7) has also been confirmed to be associated with tumor development. EMT is a cellular biological process that occurs during tumor progression and may contribute to pathological conditions such as carcinogenesis (15, 16). The progression of EMT (17, 18) involves the sequential activation of numerous intracellular signaling pathways, with the primary aim of facilitating the acquisition of a series of intermediate phenotypic states by epithelial cells along the epithelial–mesenchymal axis. These phenotypic alterations encompass changes in cancer cell motility, morphology, polarity, and function, culminating in cell stemness, enhanced invasiveness, resistance to chemotherapy and radiotherapy, and the development of cancer metastases (19). Recent reports (8, 20) indicate that the predominant cause of radioresistance in cancer is acceleration of the EMT process. Therefore, this study involved an analysis of EMT-related markers in KYSE150 and KYSE150R cells, demonstrating that EMT modulates radioresistance in esophageal cancer cells. These radioresistant esophageal cancer cells exhibited enhanced EMT characteristics. Upon silencing SATB2, the EMT characteristics of KYSE150R cells were diminished resulting in a significant inhibition of their migratory and invasive capabilities. Our findings suggested that the inhibition of SATB2 can suppress the EMT process and revert the radioresistance observed in ESCC. The Wnt/β-catenin signaling pathway was associated with radiation-induced EMT (21). Currently, studies have indicated that radiation therapy enhances Wnt signaling by upregulating the Wnt ligand expression. Typically, Wnt ligands bind to their receptor Frizzled and the co-receptor lipoprotein receptor-related protein (LRP) 5/6, thereby inhibiting GSK-3β-mediated phosphorylation of β-catenin. Concurrent radiotherapy can augment the stability of β-catenin (22). Subsequently, the stable β-catenin translocates to the nucleus, where it binds to T-cell factor (TCF)/lymphoid enhancer factor (LEF) transcription factors, thereby activating the expression of EMT-related molecules (23). Ionizing radiation (IR) also facilitates the nuclear translocation and accumulation of β-catenin, as well as enhances β-catenin/TCF transcriptional activity (24). Furthermore, the Wnt/β-catenin signaling pathway enhances the stability of the Snail protein within the nucleus by activating the Axin2 pathway, which induces EMT (25).

The Wnt/β-catenin pathway (8) is a precisely regulated signaling cascade that modulates a variety of physiological and pathological processes. Recent studies (26, 27) have underscored the involvement of the Wnt/β-catenin pathway in cancer progression and the acquisition of radioresistance in cancer cells. In cancer cells, the Wnt/β-catenin signaling pathway is upregulated, promoting EMT, a process associated with cancer invasiveness and radioresistance. The core protein of this pathway is β-catenin, which not only interacts with E-cadherin to modulate intercellular adhesion and cell migration but also functions as a pivotal downstream effector, leading to the transcriptional activation of its target gene, C-myc. It not only connects E-cadherin but also regulates cell adhesion and cell migration. The function of GSK-3β (28) is to regulate the degradation of β-catenin through autophosphorylation inactivation. Some studies have verified that GSK-3β induces radioresistance in pancreatic cancer cells via the β-catenin mechanism (29). Therefore, it is reasonable to infer that GSK-3β, β-catenin, and their target gene C-myc contribute to tumor radioresistance. As anticipated, KYSE150R cells exhibit a higher activity of Wnt/β-catenin pathway compared to their parent cell line (KYSE150 cells). Furthermore, silencing SATB2 decreases total β-catenin levels by reducing the GSK-3β (Ser9) phosphorylation, thereby weakening the EMT process. Additionally, we found that silencing SATB2 reduces the expression of the target gene C-myc by decreasing intranuclear β-catenin, thereby inhibiting cell growth. In our study, we also established a xenograft model of ESCC in BALB/C-nu/nu nude mice and found that stable low expression of SATB2 inhibits the activity of the Wnt/β-catenin signaling pathway, thus inhibiting the EMT phenotype and radioresistance of the tumor tissue, and increases apoptosis. Therefore, we concluded that the influence of SATB2 on the radioresistance of ESCC is associated with the Wnt/β-catenin signaling pathway.

## 5 Conclusion

This research demonstrates that SATB2 promotes radioresistance in ESCC. This study confirmed that SATB2 activates the Wnt/β-catenin pathway, leading to the abnormal activation of its downstream pathway, and ultimately results in EMT and radioresistance in ESCC. Therefore, SATB2 could potentially be utilized as a novel therapeutic target to combat radioresistance in ESCC.

## Data Availability

The original contributions presented in the study are included in the article/**Supplementary Material**. Further inquiries can be directed to the corresponding authors.

## Supplementary material

Supplementary Figure 1 Evaluation of Si-SATB2 knocking efficiency. Evaluation of knocking efficiency of KYSE150R cells using three different sequences of Si-SATB2.
