# Supplementary figures and images for: SATB2 promotes radiation resistance of esophageal squamous cell carcinoma by regulating epithelial-to-mesenchymal transition via the Wnt/β-catenin pathway
Source: Front Oncol. 2025 Feb 26;15:1543426. doi: 10.3389/fonc.2025.1543426 (PMC11896856; doi:10.3389/fonc.2025.1543426)

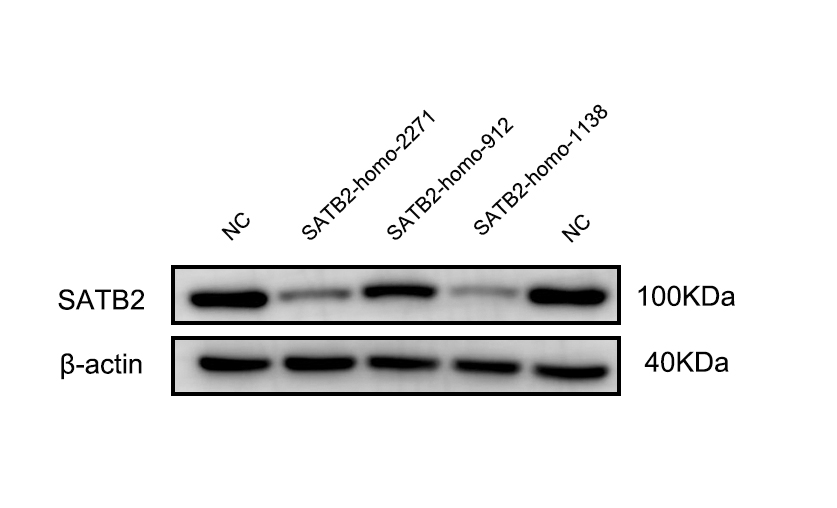

Supplement: Supplementary Figure 1 — Evaluation of Si-SATB2 knocking efficiency. Evaluation of knocking efficiency of KYSE150R cells using three different sequences of Si-SATB2. [file Image1.jpeg]
